# City to city learning and knowledge exchange for climate resilience in southern Africa

**DOI:** 10.1371/journal.pone.0227915

**Published:** 2020-01-24

**Authors:** Mzime R. Ndebele-Murisa, Chipo P. Mubaya, Lulu Pretorius, Rudo Mamombe, Kornelia Iipinge, Wilma Nchito, John K. Mfune, Gilbert Siame, Brenda Mwalukanga

**Affiliations:** 1 Department of Freshwater and Fishery Science, Chinhoyi University of Technology, Chinhoyi, Zimbabwe; 2 International Collaborations Office, Chinhoyi University of Technology, Chinhoyi, Zimbabwe; 3 School of Life Sciences, University of KwaZulu-Natal, Scottsville, Pietermaritzburg, South Africa; 4 Department of Biological Sciences, University of Namibia, Windhoek, Namibia; 5 Department of Geography and Environmental Studies, University of Zambia, Lusaka, Zambia; Curtin University, AUSTRALIA

## Abstract

Southern African cities face several challenges including management of rapid urbanization, rising populations, expanding informal settlements; adequate water and other service provision, and a host of governance challenges. Climate change and variability add a compounding effect to this complex, multi stressor context. Addressing the complexity requires an understanding of urban ecosystems functioning and interactions amongst the built and natural environment (climate) and human systems. In this paper we argue that learning is essential for cities to be resilient to current and future challenges. We profile the Future Resilience for African CiTies And Lands (FRACTAL) project which contributed towards climate resilient development by providing relevant climate information for decision-making at the city regional scale in southern Africa. Following FRACTAL’s city-to-city learning approach of sharing good practices, knowledge and experiences framed around transdisciplinary research, the study cities of Harare, Lusaka, Windhoek and Durban conducted city learning exchange visits between 2017 and 2018. We used a mixed methods approach to collect and analyze historical climate and hydrological data and current socio-economic and development data among the cities. A qualitative, in-depth, case study comparative analysis was used to identify similarities and differences as well as lessons drawn from the learning process during the city exchanges and these were complimented by desktop studies. Results showed water scarcity, large informal settlements, reliance on external water and energy sources, inadequate protection of ecologically sensitive resources and service provision as some of the common complications in the cities. Several lessons and transferable practices learnt from the cities included effective water conservation and waste management and the use of public-private partnerships in Windhoek, community engagements in Durban and Lusaka while lessons on decisive leadership in dealing with informal settlements emanated from Harare’s limited informal settlements. Lastly, Durban’s Adaptation Charter and integrated climate planning provided lessons for biodiversity protection and mainstreaming climate change at city governance level. While we recognize that cities are context-specific we consider these good practices as being broadly transferable to other southern African cities. We conclude that social, experiential and structured learning can be an innovative way of multi-stakeholder engagement and a useful approach to increase city resilience planning across southern Africa and cities that face similar developmental challenges.

## Introduction

Southern African cities, being densely populated, urban environments with expanding populations as compared to the rest of the countryside, are vulnerable to harsh weather, climate variability, extreme climate events and associated risks among other factors [1]; [2]; [3]; [4]; [5]; [6]; [7]. For instance, droughts often experienced in cities like Cape Town and Windhoek and currently across the southern African region because of low rainfall levels this past rainy season of 2018/2019 have become more frequent, intense and widespread [8]; [9]) while variations in inter annual rainfall have increased in the past half century [10]. These extreme events are projected to likely increase in the future [11]; [12]. Cities play a tripartite role when it comes to climate change as they are contributors to climate change through greenhouse gas emissions; bear the brunt of climate change; and can simultaneously be places of climate change (transformational) adaptation through innovation and solutions that can build resilience [13]; [14]. Cities in southern Africa face a multiple stressor context with several development challenges including inadequate water provision, mismanagement, governance and infrastructure challenges and lack of financial resources. At the same time, these and other cities function as ecosystems which are connected through processes that function at multi-scalar and high levels of complexity [15]; [16]. The city-space, is therefore characterized by complexity, and multiple, interconnected subsystems or ecosystems [17] in which city planning and implementation should be considered using a climate-resilience development lens. Notably, the interaction of physical ecosystems with the built environment, social and financial systems, environment and climate is indeed complex, even more so in rapidly urbanizing African city regions where intricately, interconnected infrastructure, institutions and information form the urban ecosystem. Understandably, there is growing concern about the resilience of these urban areas, particularly in vulnerable and underdeveloped regions such as southern Africa, given the connectedness and fragility of the urban ecosystems and the need for transformative over an incremental adaptation and mitigation agenda even under 1.5°C average global warming trends [16].

The multi-stressor context, which is compounded by climate change and variability, means that these cities need to respond swiftly, flexibly and creatively to develop systematic, systemic, sustainable and resilient solutions to the climate crisis. The multi-stressor context requires numerous responses, which may consist of responses in physical planning such as ‘hardening up of infrastructure systems, including storm-drainage systems, water supply and treatment plants with protective physical improvements; protection or relocation of solid waste management facilities, energy generation and distribution systems; and consolidation of hydro-geologically fragile areas’ [18], and/or ecosystem- and community-based adaptation [19] coupled with the transformative adaptation agenda given that resilient development now requires nothing less than transformational change [20]; [21]. At the same time, processes that foster adaptation, mitigation and resilience building such as learning from and adopting best practices are essential. Learning is essential for cities to adapt to current and future challenges. One approach to fostering resilience includes city to city learning in order to obtain ‘good practices’ through sharing knowledge and experiences [22]. Although Moodley [23] questions whether an international network of learning is effective where contexts and challenges are different among regions (such as southern African cities and those in the global North), he demonstrates how a structured learning process amongst cities that face similar developmental challenges can help improve the quality of urban strategic planning. It is assumed that cities that are at an advanced stage in terms of adaptation planning can act as focal points for learning to cities that are engaging in adaptation planning [24]. However, Moodley [23] demonstrated the importance of partnerships rather than mentorship as shown in a USLG program that the southern African municipalities of eThekwini (Durban) in South Africa, Otijiwarongo in Namibia, Mzuzu in Malawi were involved in. Therefore, mutual learning and equal partnerships through city networks can offer valuable lessons among cities in the continent and for promoting change using ‘early adopters’ such as the cities of Lagos and Durban [25]; [26], which have been identified as having made remarkable progress in terms of climate change adaptation [27].

In this paper we argue from the perspective that cities are connected, complex as well as vulnerable entities, which can be regarded as urban ecosystems. We aim to contribute to the argument on, and advocacy for, city to city learning. We center our discussion around the question of how useful city exchanges are in facilitating learning from experience and good practices in urban ecosystems and contributing towards building resilience in southern Africa. We draw attention to how such learning can contribute towards developing the capacity of cities in order to build climate resilience. We anticipate that lessons and good practices are relevant for a broader set of urban ecosystems that are dealing with similar environmental, climatic, demographic, institutional and developmental challenges, particularly in sub-Saharan Africa. We frame our argument by: i) outlining an overview of the various urban ecosystem issues for four southern African cities, and ii) examining the learning from each city and how it can potentially be taken on board for resilience building by the cities concerned.

### Evolution of climate research

Climate research has traditionally been conducted in siloed approaches with little to no linkages between the science, policy and society; a characteristic which has repressed the application of science for addressing societal problems [28]; [29]; [30]. The discipline-specific focus and specialization—though important—cannot address the multi-faceted challenges in society or complexities involving multiple stakeholders and interconnected urban ecosystems. Traditional approaches in climate sciences and the resilience discourse has seen climate information inappropriately regarded as a superior knowledge type [31] being developed, packaged and communicated by ‘experts’ in mostly linear trajectories that involve climate ‘producers’ and ‘end users’ [32]. This approach fails to deal with the multiple stressor context and interconnected, complex urban ecosystems. In addition, a host of information is ‘lost’ that helps to contextualize the environment of the end-user [33]. There is a need to move beyond linear approaches to communicating climate information to ensure closer alignment with end-user needs, based on a growing demand for more usable climate information as societies deal with its real and devastating cross-cutting impacts.

There is a growing awareness that new ways of knowledge production and decision-making must move beyond disciplinary silos, and that approaches such as transdisciplinary research, and knowledge co-production may provide the means to do so [34]; [35]; [36]. Disciplinary silos compartmentalize knowledge and emphasize introverted specialization while transdisciplinary research incorporates different actors, disciplines and knowledge bases from science and practice [37], and has the potential to close the gap between knowledge and action- making science relevant for addressing climate change adaptation and building resilience [38]; [39]. In the transdisciplinary research approach, where stakeholder engagement and inclusivity is encouraged, reflexivity around concept framing, inclusion of perspectives, knowledge, and experiences, a better understanding of how communication affects interpretation and trust, and mechanisms of knowledge exchange and communication of climate information can be used more effectively toward producing climate solutions and responses [40]; [41]. The inclusive and participatory approach in transdisciplinarity works well as it seeks to address challenges associated with complex urban ecosystems [42].

### Social and experiential learning

The concept of learning is based on the premise that change occurs across spatial and temporal scales and we argue that the interconnectedness of socio-ecological systems presents the need to increase our global cognitive, behavioural and moral intelligence through learning [43]. This brings to the fore, the concept of social learning (learning from each other) which transcends the local scale by crossing boundaries such as cities and countries [44]; [45]; [46]. Wenger [47]) puts emphasis on how learning can be used to make better decisions in the future. In that regard, Tschakert and Dietrich [48] emphasize anticipatory learning for climate change adaptation which is learning that is forward-looking. All these concepts point to building and developing resilience as well as partnerships [49]; [50]; [51]. Approaches to learning are embedded in the social learning theory which explains why people behave the way they do based on intrinsic and extrinsic, and especially social influence [52]. This places social learning within a cognitive decision-making framework [53] as well as the knowledge sharing mantra to promote application of knowledge. Our approach to social learning follows Bandura’s first proponent, which is learning from others through observation in the hope that this may lead to the rest of the social learning components of application, i.e. imitation and modeling. Weissberg and Cascarino [54] define social (and emotional) learning as comprising five sequential elements which are self-awareness, self-management, social awareness, relational skills and responsible decision-making. Social learning provides knowledge that is essential for institutional capacity building and sustainable development [55]. Decision-making is a major component in the steering of sustainable development and resilience and provides a platform where both lessons can be drawn from, and where implementation of lessons for tailor-made development based on the understanding of the context and decision-making processes, can be derived. Transformative decision-making processes which promote integrated planning are necessarily for resilience building and these processes are fostered by processes of learning and imbued under concepts such as leadership and strategies (see ARUP’s city resilience framework).

The importance of knowledge sharing, and cross city learning is evidenced by the emergence of city networks such as the C40 Cities Climate Leadership Group, ICLEI- Local Governments for Sustainability and the international United Cities and Local Governments (UCLG) network [44]; [45]; [46]; [23]; [24]. New approaches to research in the last couple of decades have fostered the concept of learning in a bid to encourage the addressing of complex global challenges such as climate change and variability in urban and other ecosystems. The importance of learning in dealing with complex challenges can be drawn from Wenger ([47] pp 3) who posits that, *what if we assumed that learning is as much a part of our human nature as eating and sleeping*, *that is both life-sustaining and inevitable*? The co-development of new knowledge generated through collaborative, transdisciplinary research efforts, fosters diverse ways of learning, including the concept of experiential ‘learning as you go’ which implies iterative, reflective and feedback mechanisms. The use of these learning concepts have been on the rise, given funders’ recent (in the past decade) inclination towards larger-scale, impactful research that solves complex problems; versus the traditional and mere generation of new knowledge and the need for socially robust knowledge in adapting to climate change and building resilience [49]; [50]; [51]. Knowledge generation and sharing, as well as learning for implementation, have been emphasized as some of the important components of successful research, while underscoring the importance of learning from past experiences and scaling-up of successful models of adaptation and resilience approaches which encompass components of experiential learning [52]; [33].

### Contextualizing city learning within a multi-country research project; the case of FRACTAL

The Future Climate for African Cities And Lands (FRACTAL) research project employed and emphasized the use of city learning and contextualizing of issues, by understanding decision-making processes in cities as social learning concepts and in a bid to help build resilience through informed decision-making in the project’s study cities. This research project, which ran for a four-year period from 2015 to 2019; operated in nine cities in southern Africa, gave the participating cities impetus to learn together using a city-to-city or cross-city learning approach, and thus provided the basis for this paper. The specific goal of FRACTAL was to advance scientific knowledge of the southern African regional climate responses to human drivers of climate change and enhance the integration of scientific climate knowledge into city regional decision-making for building resilience [42]. This was done by providing city-scale, co-produced, relevant and useful climate information for study cities in the region to help develop resilience through transdisciplinary research, which learns from and informs decision-making. The focus is on university officials partnering with city practitioners in the process of formulating research questions framed around resilience, co-exploring solutions, and disseminating and presenting the findings within a transdisciplinary space, which allows time and space to disagree, debate, test alternatives and learn from mistakes; which in essence is experiential learning. The transdisciplinary research approach in FRACTAL has been operationalized through a number of processes, such as a series of multi-city ‘learning labs’; the use of embedded researchers (ERs) as bridges between academia and city municipality administrations; as well as learning exchanges between multiple cities. It is this last approach which is the focus of our paper, and which outlines the processes and outcomes of this innovative way of research in multi-stakeholder engagement as an approach to increase learning, city resilience and improved urban planning.

The FRACTAL project is hinged on four interconnected concepts and practices (transdisciplinary co-production (of knowledge), distillation (of climate information), receptivity and capacity development), which guide the learning and knowledge production as well as sharing processes. The aim of these concepts is to expand and (semi) formalize spaces of connection and co-production to be more inclusive, diverse and consequential; develop capacity to engage and know more, differently; develop receptivity to exercise agency in co-producing knowledge and decisions; and distill information through bringing it to bear on decisions [56]. Although the city exchange visits were conducted mainly to encourage and facilitate the transdisciplinary knowledge co-production concept [57]; the purposes of the other three concepts were also served well. During the city exchange visits, researchers, decision makers and practitioners from two or more cities shared experiences and learnt from each other about responding and adapting to (distilled) climate information, decision-making, city contexts and key issues as well as solutions. In the process, receptivity was created to new, innovative, climate change action-related ideas and approaches in other cities, with the aim of advancing capacity to respond likewise in their own contexts.

#### FRACTAL city learning approach

The FRACTAL city learning process was founded on the understanding that cities function as complex ecosystems and that the learning component is an important facet for engaging city decision makers and practitioners, among other facets, such as the development of climate services information. In the learning process, city labs were used as an important platform for promoting dialogue and learning, through which the exploration of solutions, drawing on both research and practice and the expertise of participants was done. The labs provided a space where academia, civil society, city and government officials interacted, co-identified and prioritized projects, mapped out problems and co- produced potential solutions to prioritized projects within the cities. The learning-by-doing and engagement approaches were emphasized in the labs [32]. In this approach, participants recognized that learning is not limited to individual city stakeholders, but rather, that the learning fora should be extended to other FRACTAL cities that exhibit similar issues or have innovations that another city could benefit and learn from.

Following this approach, the FRACTAL city learning visits were co-designed to ensure that cross-learning from experiences, lessons, best practices and failures which were drawn by a wide range of participants from the visiting and visited city were demonstrated. The goal of the exchange visits was to encourage inter-city learning and to strengthen relationships and foster partnerships, as well as contribute towards capacity development given that challenges faced by southern African cities can be similar. Another aim of the exchange visits was to provide a visual of the risks faced and processes being undertaken in the respective cities, and to share lessons from good practices (and failures) through specific site visits. The idea was that visitors would then take the learning and experiences back to their cities and institutions for further knowledge sharing as well as championing the implementation of some of the aspects learnt.

In addition, the city learning exchanges further provided the opportunity to co-identify similar problems across the FRACTAL cities participating in the exchange visits, co-develop solutions and cross pollinate on viable decisions that could be made to build resilience to the effects of climate change in cities. Co-production of outputs was also promoted and facilitated as the learnings were disseminated through various media such as blogs and reports that were co-authored by the city officials, early career and embedded researchers and local university academics from the cities involved in the learning exchanges. In all the city exchanges, emphasis was placed on a systems approach in understanding context, values, and city-specific issues while social and experiential learning and building relationships and equal partnerships stood out as major themes. This is important in the context of the (southern) African region, where mostly top-down concepts have been imposed without understanding and considering the local context in intervention program designs.

## Materials and methods

**Fig 1:**
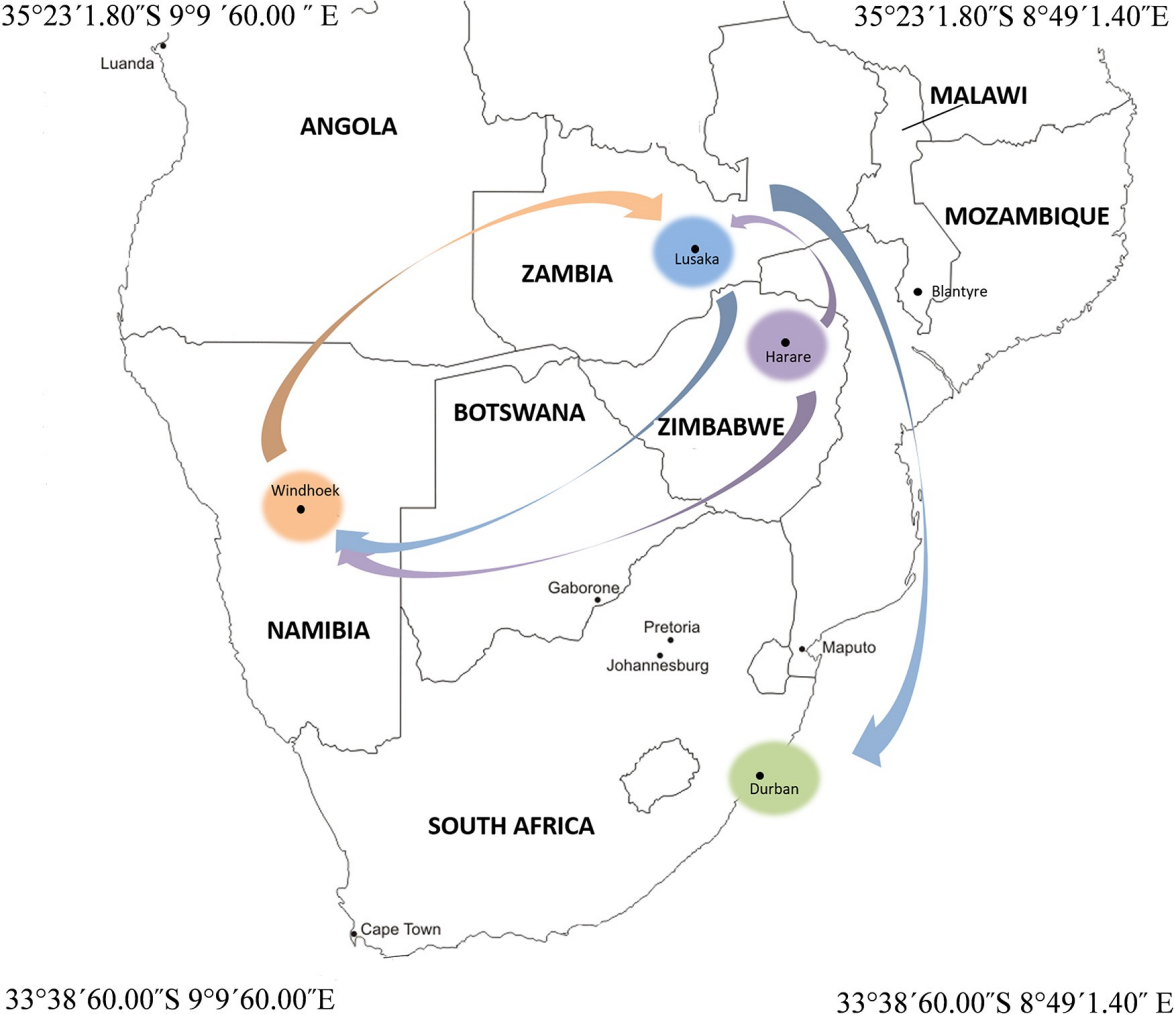
An illustration of city exchanges across southern Africa; Blue- Lusaka in Zambia, Purple- Harare in Zimbabwe, Orange- Windhoek in Namibia and Green- Durban in South Africa. The arrows indicate direction of exchange.

Between August 2017 and February 2018, the cities of Harare, Windhoek, Lusaka and Durban conducted learning exchanges through START International’s Global Environmental Change (GEC) Research in Africa and Small Opportunity Grants (SOGs) under the FRACTAL project. The visits brought together key city actors and academic researchers to share knowledge and experiences related to the on-going water (energy) and climate change work in the cities of Harare (Zimbabwe), Windhoek (Namibia), Lusaka (Zambia) and Durban (South Africa) (see Fig 1 for exchange visit routes).

A mixed methods approach was used to collect data from the study cities and to determine both climate and non- climate risks and vulnerabilities, as well as city infrastructure and governance systems. Both quantitative and qualitative research methods were employed. For the quantitative analysis, 30 to 50 years of historical climate and hydrological data from the cities’ water sources- the river catchments were collected (Table 1). This data was used to determine historical climate and hydrological trends using time series analysis. For the qualitative data collection, household surveys, key informant interviews (KIIs) and focus group discussions (FDGs) were used. A total of 1483 of both open and closed household questionnaires were administered across the cities mostly in informal settlements with the exception of Durban where no questionnaires were distributed and Harare where a cross sectional representative sample across high, medium and low density suburbs were interviewed (Table 1 and S1–S10 Files). Questionnaires and KIIs gathered socio-economic and demographic data as well as perceptions on climate and resilience which were recorded by enumerators. Additionally, facilitators and embedded researchers recorded responses on climate perceptions using FDGs, during workshops and city learning labs. Key informants and FGD interviews and workshops as well as city labs discussions were framed around key issues following the FRACTAL engagement approach which incorporated interactive methods such as games to foster engagement from a pool of diverse city stakeholders and participants. The city learning labs and dialogues were facilitated as either initial consultative and/or project validation workshops (Table 1). These engagement processes in the cities collated information, which was documented as interview transcriptions, reports and in the case of the city of Lusaka, flood maps.

**Table 1:** Sources of information in the cities.

City (year)	Sampling details
*Historical climate and hydrological data*	
Harare (1984–2014)	30 years monthly rainfall totals and temperature averages at 3 weather stations (Belvedere, Chivero and Kutsaga) data was obtained from Zimbabwe Met services Department as well as Upper Manyame River flows from eight (8) gauging stations (C2, C3, C17, C21, C22, C24, C83 and C89) obtained from ZINWA
Lusaka (1967–2017)	50 years rainy season (November to April) rainfall totals and standard precipitation index for the period 1967 to 2017 was collected from the Met services for Lusaka. A 12.5m resolution Digital Elevation Model (DEM) from ALOS PALSAR in ArcMap was used to analyze flooding using height above the nearest channel base
Windhoek (1977–2017)	40 years monthly rainfall totals were collected from Okahandja and Windhoek Stations and Meteorological Office in Windhoek. Streamflow for the Swakop River in the Swakop-Omaruru and Omatako-Okavango catchments as well as three (3) dams (Swakoppoort, Omatako and Von Bach) water levels and groundwater table levels
*Household questionnaires*	
Harare (2017)	120 respondents from six suburbs (Mabvuku, Stoneridge, Mainway Meadows, Marlborough, Mt. Pleasant Heights and Vinona) representing 3 density levels (high, medium and low and old versus new settlements)
Lusaka (2017)	500 respondents from two informal settlements (Kalikiliki and Kanyama)
Windhoek (2017–2018)	863 respondents from across the city (John Pandeni, Katutura Central, Katutura East, Khomasdal, Moses Garoeb, Samora Machel, Tobias Hainyeko, Windhoek Rural and Windhoek West) with the largest (~60%) representation from informal settlements
*Key informant interviews*	
Harare (2017)	18 key informants from several organizations: Harare City Council; Ministry of Environment, Water and Climate; climate change policy coordinators; and civil society
Lusaka (2017)	14 key informants (9 from SOG and 5 from GEC) from LuWSI, Lusaka City Council, GiZ, National water and sanitation council, Lusaka Water and Sewerage Company and Water Resources Management Authority
Windhoek (2017–2018)	4 key informants from UJaams Water Reclamation Plant, MeatCo and City of Windhoek
*Workshops*	
Harare	2- Project initial consultation and culmination (validation)
Lusaka	2- Stakeholder engagement and project culmination
Windhoek	1- Stakeholder workshop- dissemination of findings
*Document and policy review*	
Durban (2014)	Durban Adaptation Charter (DAC); South Africa National Climate Change Response and the mitigation Policy
Harare (2016–2019)	Zimbabwe National Water Policy, Climate Change Policy; Climate change response and strategic Plan; Harare Masterplan
Lusaka (2016–2019)	Lusaka Water Security Initiative (LuWSI); Zambia National Climate Policy, Water Resources Management Act 2011, Zambia National Water Policy

After the field and data collection was done in each city, city exchange visits were organized in such a way that researchers from visiting cities could learn about the host cities’ sources of information, sampling techniques, relevant sites, policies and practices using field and site visits. Visiting and hosting city teams held joint community and council meetings in the host cities and discussed commonalities in thematic problems as well as potential solutions using guided agenda (see S8–S10 Files). Simultaneous to the field sampling and exchange visits, desktop studies were used to complement the learning labs and engagements that we convened in the cities and collectively helped to identify key issues for the study cities and urban ecosystems, and to assess the cities’ advancement and gaps that need to be addressed in resilience planning. Altogether, the mixed methods approach of collecting data where we used multiple (> three- 3) approaches, sources and types of information including during the city exchange visits allowed triangulation and verification of trends, perceptions and assessments in order to reduce bias that emanates from single informants, methods, observers or single theory studies [58]. Prior to official engagement, participants were informed of the FRACTAL and follow-on projects through preliminary meetings and during data collection cover letters and introductory statements were used to inform participants about the study. No identifiable information was collected from the participants in the surveys so that data was collected anonymously. We therefore adhered to principles and practices of ethical research which included among others, voluntary participation, informed consent, respect for people and communities, confidentiality and anonymity. Simultaneously, desktop studies (literature review, document and policy analyses) were conducted to review policies and city developmental plans (Table 1).

The collated data on the cities is unpacked in the results section of this paper. In response to the question on the usefulness of city learning in contributing to building resilience that we pose in the introduction; we conducted a comparative case study analysis. Given the disparity in the type of data collected across the cities which was due to the limited scope of the study in undertaking an experimental design across the cities, a comparative, in-depth case study analysis was relevant for evaluating and comparing the condition(s) of the cities. In the comparative analysis, we focused on the cities’ advancements in their responses and adaptation to climate change, particularly for water conservation, energy, solid waste management and biodiversity protection mobilized against risks and vulnerabilities as a result of climate-induced and non-climatic factors (see Table 2 for specific purpose of visits). The case study approach is appropriate for analysing and synthesizing ‘similarities, differences and patterns across two or more cases… and understanding how the context influences the success of an intervention’ ([59] pp 1). Thus, this approach provided the opportunity for us to single out best- and worst-case scenarios and in this way draw out lessons, good practices, failures and to evaluate the process of learning in the context of climate resilience across the cities. This effectively helped to answer the research questions on outlining issues in the study cities including good practices and examining the usefulness of city learning across the region for building resilience.

**Table 2:** Summary of key issues, city exchange visits and visit purpose.

City	Key issues	Host city	Purpose
**Harare**	Water supply challenges Low quality water Poor solid waste management Wetlands degradation Integration of climate change into planning at city level	Lusaka and Windhoek	Understand adaptation strategies Compare risks and vulnerabilities in the water and energy sectors
**Lusaka**	Flooding Low quality water Water insecurity Poor solid waste management Integration of climate change into planning at city level Informal settlements	Windhoek and Durban	Understand adaptive measures Suggest recommendations for increasing awareness and adaptation capacity Promote sustainable use and protection of surface and ground water sources in Lusaka
**Durban**	Flooding Informal settlements	(Hosted Lusaka delegation)	Examine LuWSI and the water security initiatives being implemented for coordination Develop quick win projects that can be delivered in a short time frame
**Windhoek**	Water supply challenges Low quantity water Rural-urban migration Informal settlements	Lusaka	Bring key city actors together Share knowledge and experiences related to the ongoing water and climate change work in both cities

## Results

### Key issues in the urban ecosystems

A suite of risks that are impeding sound service delivery in the four cities were identified in the workshops and city learning labs during the identification of city key issues (see Table 2). Chief among these were water security as a key risk of climate change. We also identified dynamics of water and solid waste; informal settlements; energy; water governance; and climate change planning and policy integration as presenting both problems and solutions across the cities. We present brief descriptions of the cities and summaries of key issues identified through desktop studies and during the city exchanges in order to provide city-specific contexts and as obtained from the sources of data shown in Table 1.

#### Harare

The Zimbabwean capital is situated in the north-eastern part of the country. Inadequate supply of potable water was a key issue for the city. Zimbabwe is divided into seven river catchments as a way of decentralizing water provision. Harare falls under the Upper Manyame Catchment with Lake Chivero, an artificial reservoir that was built in the 1950s situated downstream of the city, being the city’s main raw water source. The Zimbabwe National Water Authority (ZINWA) is responsible for maintenance of all surface water bodies such as dams as well as underground water resources across the country, whilst city councils such as Harare City Council (HCC) are responsible for provision of potable water to all residents as stipulated by the Urban Councils Acts. HCC purchases raw water from ZINWA and then treats and distributes it to residents and industries. It is noteworthy that the two entities (ZINWA and HCC) are housed under different ministries and this presents challenges in the governance of and provision of water for the city. ZINWA is housed under the Ministry of Lands, Agriculture, Water, Climate and Rural settlement whereas HCC is housed under the Ministry of Local Government and National Housing.

With a population of 1.6 (2.8 in the metropolitan area) million, Harare is the industrial hub of the country. During the co-design processes of the FRACTAL project, stakeholders identified potable water supply as the major key issue followed by wetlands degradation and water governance (see Table 2) showing that there are grave concerns around issues to do with water in the city. Urban agriculture, which draws a substantial amount of water for production through irrigation particularly in the form of horticulture is practiced to a large extent in and around the city. The city has aged water and sanitation infrastructure which is expensive to maintain. Because the city is located upstream of its catchment, partially treated wastewater, together with pollutants from industries and agriculture ends up being washed into the main water source for the city, Lake Chivero [60]. Climate-induced impacts such as reduced availability of rainwater during drought and dry spells or in the opposing and extreme case, flooding exacerbate challenges of access to safe water, sanitation and hygiene and further lessen the city’s capacity to provide potable water, given its already limited capacity.

Contested development in an almost unregulated manner is leading to the degradation of wetlands in the city. This is a real challenge as it threatens the city’s water catchment processes. Another challenge in Harare and the nation is of energy supply which, like raw water is generated from external sources far from the city and is also distributed at national and not at city level. There is a nexus between climate (rainfall), water and energy which is mostly wood biomass, followed by hydropower, thermal and coal-based forms which all use water in their production. Despite a relatively high electrification rate (45%) in comparison to other SADC countries, current power supplies are inadequate for meeting energy demand across the country and of late the city and country have been subject to significant power load shedding which negatively affects productivity. In the past decade, both fuel and electricity shortages have hit the country partly as a result of recurrent droughts negatively affecting hydropower production and also as a result of forex and budget constraints for purchasing fuel and electricity from neighbouring countries with excess power such as Mozambique and South Africa.

#### Windhoek

Namibia, like many countries in semi-arid areas, is characterized by high climatic variability in the form of persistent droughts, unpredictable and variable rainfall patterns leading to scarcity of water [61]. Coupled with rapid urbanization, the threats of persistent water scarcity are real. A growing concern is the city of Windhoek, with a population of approximately 268,000 and an urbanization rate of 3.1% per annum. This surge in the population has created many problems for urban planning and vulnerabilities such as water shortage, energy problems, poor sanitation and waste management and related health problems. The city of Windhoek is charged with the responsibility for urban planning but is constrained or challenged by the high influx of people from rural areas. Among others, the persistent droughts and unpredictable and variable rainfall patterns, due in part to climate change, adversely affects the availability and supply of acceptable quantities and quality of water to the city of Windhoek (Table 2). This is exacerbated by the high rate of urbanization. Hence, there is a strain on service delivery, including water supply, land and infrastructural development.

These issues are compounded by low quantity water available for domestic use for the city residents, and energy supply challenges. The situation is made worse by informal settlements, which tend to be fueled by rural-urban migrations that have led to increased populations in the city. Most informal structures in Windhoek are made of corrugated sheets, which the local authorities tolerate as these are temporary structures that can easily be removed while the residents in the informal settlements rely mostly on wood for energy and lack access to proper sanitation facilities.

#### Lusaka

The City of Lusaka’s 1.7 million inhabitants face significant climate related challenges such as urban flooding on a yearly basis (see Table 2). Geological factors largely contribute to flooding in the city but flood risk in Lusaka appears to be exacerbated by poor municipal solid waste management systems [62]. Large parts of the city are underlain by limestone, which is porous, creating underground aquifers which easily become saturated. This in turn creates a high water-table and results in flooding when the city experiences high rainfall. The water table fluctuations do not exceed 5 metres and during the rainy season borehole water tables reach ground surface [63].

Informal settlements in the city tend to compound the multi-stressor context for Lusaka. This is because the major aerial extent (70%) of the city has developed informally and lacks proper structures to drain excess water [64]. Another major issue is the management of solid waste—garbage that is left uncollected which ends up blocking the drainage systems. Urban planners and city decision-makers do not understand how municipal solid waste interacts with climate change, infrastructure, and urban planning to affect urban flooding and flood risk (Table 2).

#### Durban

Major flooding risks as a result of increased variability in rainfall and extreme storm events under climate change is one of the challenges facing the city of Durban (see Table 2). Durban is a middle-income African city of 3.5 million people residing under highly unequal social, economic and environmental conditions—a legacy inherited from the Apartheid regime. However, what is notable for this city is significant strides that have been made towards the integration of climate change into planning at city level and evidence on the ground of projects and efforts to deal with the potential challenges that were identified. The anticipation that climate change could undermine development efforts and exacerbate the plight of the city’s most vulnerable residents in informal settlements gave the eThekwini Municipality the impetus for innovative adaptation efforts. More recently, the city has been hit by a series of floods which has exposed its infrastructure and more so the informal settlements.

### City learning points

The multiple stressor context and interconnectedness of the urban ecosystem across the four study cities of Harare, Windhoek, Lusaka and Durban was demonstrated well by presenting similarities and differences (see Tables 3 and 4). We present the learning points in five emerging thematic areas, namely water and waste, energy, informal settlements, water governance and city climate integration and planning.

**Table 3:** Similarities across the study cities.

Similarities	Cities
Income groups determined levels of risks and vulnerabilities	Durban, Harare, Lusaka, Windhoek
Multiple stressor context; climatic and non-climatic risks	
High levels of informal settlements	Durban, Lusaka, Windhoek
Droughts	Harare, Lusaka, Windhoek
Flooding	Durban, Lusaka, Harare
Unpredictable rainfall patterns	Harare, Lusaka, Windhoek
Low quality and inadequate potable water supply and solid waste disposal; unregulated water abstraction	Harare and Lusaka
Comparable renewable energy efforts	Harare and Windhoek

**Table 4:** Differences across the study cities.

Differences	Cities
*Informal settlements*: High to low levels of un- to under-serviced households	Lusaka -70%, Windhoek—45%, Durban—28%, Harare -9 to 13%
*Water scarcity*: Highest in Windhoek, then Harare and Lusaka	Harare, Lusaka and Windhoek
*Water supply*: Controlled by public entities	Harare and Durban
Managed by a private company	Lusaka and Windhoek
*Energy supply*: Mostly coal driven	Durban
Mostly biomass (wood) then hydro-powered	Harare, Lusaka, Windhoek Harare
City-specific	Windhoek
Controlled by public entities	Durban, Harare, Lusaka
*City climate integration and planning*: Climate change integrated in city climate change strategy	Durban
Advanced stages to integrate climate policy and action from national level to local government level planning	Windhoek
Still to develop Climate Change Strategy and Action plan	Harare and Lusaka

#### Water and waste issues

As a learning point, the City of Windhoek (CoW) is implementing efficient water conservation measures, which are linked to several factors and actions that form part of the city’s water ecosystem. These factors include climate (droughts), raw water availability, water scarcity and/or availability of water from remote catchment areas. The city deals with water scarcity through several initiatives such as wastewater reclamation and desalinization (although this is not a specific CoW initiative), artificial recharge of aquifers, water rationing and awareness campaigns to change water use behavior e.g. changing gardens by replacing water intensive plants with indigenous alternatives that are drought tolerant and allowing only public green spaces to be watered and this through wastewater irrigation [65]. More raw water is available from Harare city’s river catchment, where the Greater Harare area receives an average of more than 700mm of rainfall per rainy season as compared to Windhoek’s paltry 365mm and where very high mean evaporation rates (3,400mm) per year exist.

Another issue is the reliance of these cities like Lusaka and CoW, on external and far water sources namely Kafue, Omatako, Swakoppoort and Von Bach dams located some 60 km to 200 km away from the cities; and Kafue River where several projects have and continue to take place to increase abstraction for Lusaka. Climate-induced flash floods and intense rainfall coupled with inadequate stormwater infrastructure result in health implications that are associated with unhealthy, sanitary conditions and affect vulnerable communities such as in Kanyama and Kalikiliki informal settlements of Lusaka. While Harare and Lusaka are also challenged with solid waste management, CoW in contrast has a reliable solid waste management plan and it implements ward contractor systems in informal settlements where waste is picked up weekly at households and business with daily clean-up of open spaces. This is a good practice that many African cities which also have high informal settlements can learn from as the cities struggle with solid waste disposal management of which the city of Harare, once called the ‘sunshine city’ because of its historic efficacious waste management systems from decades ago, is no exception. Harare and Lusaka municipalities, which can learn from CoW, appear to have failed to effectively deal with solid waste as well as waste water treatment as over the years, increased mushrooming of informal dumping sites and decomposed piles of garbage now characterize the cities; broken sewer pipes discharge raw waste into clean water sources and the provision of clean water is still way below the expected standards.

Lessons were also learnt on waste management from Durban, which has numerous fully engineered landfill sites; one of which is the award-winning site at Buffelsdraai. The land reclamation in the buffer zone of the Buffelsdraai landfill through planting of indigenous trees, is an example of climate adaptation and mitigation being practiced by the local authorities; through green job creation, carbon sequestration, and land restoration. The tree planting project in Durban which uses ‘treepreneurs’ in a Community Reforestation Project presented a viable option to planting trees around the city of Lusaka to increase the green spaces in the city. The Sihlanzimvelo Programme, which uses local community businesses to deal with the issue of waste and alien plants causing flooding of streams and rivers by blocking the waterways during flood events, was another learning opportunity from Durban for Lusaka.

The city of Durban also illustrated the importance of biodiversity and ecological ‘green’ infrastructure as part of the urban ecosystem; and how the incorporation of this bioresource into city planning is important for building resilience and as part of the climate action response. This lesson is important for other southern African/African cities where bioresource conservation is often not a priority because city planners and decision-makers may not view the city as an ecosystem. In contrast to Durban, the city of Harare appears to be failing in the area of biodiversity conservation by allowing development in and around its wetlands [66]. Developments on wetlands in the past two decades are leading to less groundwater and throughflow recharge to the city’s rivers and eventually the reservoirs as wetlands are a major source of water for the city [67]. Despite the importance of these wetlands and their function as part of the city’s river recharge system and therefore the need for conservation measures [68], there has been unprecedented encroachment and degradation of wetlands across the city of Harare. This has posed a threat to the delicate wetland ecosystems and subsequent services and in this case the availability of raw and clean water for the city’s use.

#### Informal settlements

The exchange visits to Lusaka and Windhoek; and illustrated to a lesser extent in Durban; showed high proportions of informal settlements in the cities with the exception of Harare which has a low percentage coverage of informal settlements (Table 4). A notable difference in the informal settlements in the various cities is the permanency of the housing structures and its contribution to exacerbated climate impacts such as flooding. An example is Windhoek’s informal settlements, which are made up of temporary structures, whilst the structures in Lusaka are permanent. In Lusaka, the issue of flooding and solid waste disposal is a huge problem in informal settlements such as in Kanyama (one of the city exchanges sites); where only a tenth of the over 300,000 residents have access to potable water. Lusaka stands out, as it has a long history of permanent, unplanned and illegal settlements. In Lusaka, the proliferation of permanent illegal structures makes upgrading through redevelopment and provision of services costly and difficult to attain. Services such as waste management cannot be provided to these areas using conventional methods, therefore community-based options have been resorted to. Unfortunately, these Community Based Enterprises (CBEs) lack capacity and know-how, causing waste to pile up and subsequently contribute to flooding in some of these settlements. The lesson learnt from Katutura in Windhoek was the proactive actions taken by CoW in curtailing the expansion of the unplanned settlement by putting up fencing and allowing only temporary structures.

In comparison, Williams et al. [69] demonstrated how the Palmiet Rehabilitation Project in Durban, a multi sector partnership helped develop a deeper understanding of the current governance system, and embedded social values enacted in the case of the Quarry Road West informal settlement. This was helpful for integrative and transdisciplinary management of flood risk, at the climate change and water governance interface as climate change adaptation remains a challenge for decision-makers and policy-planners in the city. The need for engagement and more effective integration of informal settlements into local governance for water management was demonstrated lately, when informal settlements, including Quarry Road in Durban were hard-hit by the recent flooding (April 2019) that caused damage to infrastructure. Reports projected cost of repairs for damaged stormwater pipes, washed away walls and culvert and subsequent repairs related to human settlements to be over hundreds of thousands of dollars including repairs to reconstruction and development program (RDP) houses, transit camp units, informal settlements, retaining walls and hostel blocks as well as costs related with electricity repairs to eleven substations.

Only one out of Harare’s 33 wards is an informal settlement and plans to formalize this area are advanced. Here, very strict infrastructure and by-laws and standards by the city are applied and enforced. This has seen the city destroying informal structures through an initiative dubbed ‘Operation *Murambatsvina* ("Drive Out Trash"), which started in 2005 and has been used by HCC in a bid to curb criminality and disease. Over the years, approaches to deal with informal settlements have also included formalization of informal settlements such as the famous Epworth and Churu Farm in Harare, coupled with building initiatives such as Operation *Garikayi/Hlalani Kuhle* (Operation "Better Living"), which consists of building concrete housing. These efforts have led to limited informal settlements in Harare and the urban areas of Zimbabwe. A positive development was noted in that there are deliberate efforts to formalize informal settlements in the city of Windhoek.

#### Energy issues

Currently, the Electricity Centre Board (ECB), regulates electricity distributors in Windhoek and other regions and there is some co-dependence in terms of energy supply to the city (Table 3). The major power plant is in the North of the country as well as the conflicts with Botswana over the use of the Okavango for water and energy supply. CoW buys energy from NamPower and supplies electricity to the residents directly whilst this is done at national level by South Africa (Eskom), Zambia (ZESCO) and in Zimbabwe through ZEDC, a subsidiary company of the Zimbabwe Electricity Supply Association (ZESA). ZEDC is solely responsible for energy supply and regulation of rates in Harare and Zimbabwe from the main grid.

South Africa is ranked 114th out of 115 nations in its readiness for an energy transition away from fossil fuels [70], and yet addressing climate issues and associated problems in the region is closely related to tackling southern Africa’s dependence on coal. Climate change poses a looming power threat to hydropower, with changing rainfall, temperature and evaporation in watersheds such as the Zambezi (where countries such as Zimbabwe, Zambia and other southern African countries and subsequent cities draw hydropower from) have potential impacts on hydropower production. Power rationing programs have been implemented across the region, including in South African and Zimbabwean cities. Lusaka and Harare have a common energy production and supply resource having a 45:55% power sharing between Zambia and Zimbabwe respectively from Kariba hydropower stations that are along the Zambezi River, but Zambia also draws hydropower along the Kafue River.

#### Water governance

The CoW provides an admirable example of how partnerships—in this case, a public-private-partnership (PPP) has worked in favour of the city. Windhoek is the only city among the four, which reclaims domestic wastewater to potable drinking water standards at the Goreangab Water Reclamation plant, which was one of the sites visited under the city exchanges. The reclamation process began operations in 1968, when the plant was built by the CoW to reclaim waste directly from domestic sewage effluent [71]. The new Goreangab Water Reclamation plant was built in 2002 and has been based on extensive experience, local research, and input from international experts to assure compliance to the strictest water quality guidelines applied internationally [72]. The Resilience City Planning Framework (RCPF) is a complex phenomenon, non-deterministic, dynamic in structure, and uncertain in nature [73]. This context poses new opportunities for collaboration among public, private, civil institutions and organizations on all levels.

In addition, artificial recharge of aquifers implemented in the CoW provides another important lesson in groundwater abstraction and water management. The city supplies groundwater abstracted from municipal production boreholes, which is different from Harare, where water management is under the control of public entities, and water treatment and supply is the sole mandate of the city council. In Lusaka on the other hand, water is managed by a commercial utility, which is a private company wholly owned by the local authority [74]. Surface and groundwater resources in Harare and Lusaka are dwindling due to increase in demand and unregulated abstraction. In Harare, drilling a borehole requires a permit from the country’s raw water regulators, the Zimbabwe National Water Authority (ZINWA) but they are poor regulators and enforcers of this piece of legislation. As such individuals across Harare have drilled boreholes in their residences, in a similar way to Lusaka. In Lusaka, borehole drilling has only recently been regulated by the Water Resources Management Agency (WARMA), which issues permits for abstraction. The Water Resources Management Act Number 21 of 2011 requires all borehole owners to register their boreholes at a fee. This is being done in order to take stock of the number of boreholes and to monitor the abstraction levels of groundwater, which is at risk of contamination and depletion in Lusaka. In contrast, in Windhoek, permission for borehole abstraction is granted by the Ministry of Agriculture, Water and Forestry (MAWF), and the city has created an efficient groundwater abstraction and recharge system where water can be artificially recharged into city aquifers for storage purposes, since the evaporation rates are very high. The same water is treated to potable water standards before it is pumped into permissible boreholes in a scheme known as the Windhoek Managed Aquifer Recharge Scheme, providing a centralized borehole system which is easier to monitor and maintain; which again provides a good case of water management that other cities can learn from.

Another interesting difference was noted in water governance structures in Windhoek City which not only buys water from Namibia Water Corporation (NamWater), but also energy from NamPower and then sells these as services to residents but under government regulated rates. In contrast, ZINWA is responsible for overall management of all raw water forms in Zimbabwe including all cities. Harare City is solely responsible for the treatment, supply and therefore charging and collection of rates for potable water and purchases water from the national distributor, then redistributes at a slightly higher cost as part of the revenue collection. The Zambian situation presents a different scenario where water in Lusaka is supplied by a Commercial Utility (CU)- Lusaka Water and Sewerage Company (LWSC), which was created in 1988 although it only started functioning in 1990. Initially, water was supplied by the local authority which lacked capacity and could not attract professionals. The CU is responsible for the abstraction (from both surface and ground sources), bulk transportation, treatment and supply of water for which it charges fees under a regulated tariff structure Water reforms of 1997 created the National Water and Sanitation Council (NWASCO), which regulates the provision of water supply and sanitation services country wide. NWASCO regulates tariffs therefore the CU is not able to have its own fee structure for water.

Currently, Lusaka city cannot be said to have inadequate water, but the growing population and the expansion of unplanned settlements compound the difficulties currently encountered in the provision of water and sanitation services. Currently, only about 30% of the city is supplied with water by LWSC, which is serviced by a sewer network. Ninety percent (90%) of unplanned households use pit latrine toilets; 3% have no toilet facilities and use bush toilets. Pit latrines are shared among several households, and this reduces the latrine’s life span and because of inaccessibility to the unplanned settlements suction tankers cannot empty the majority of pit latrines. Local boreholes are unprotected, resulting in severe health risks when there is contamination from pit latrines.

#### City climate integration and planning

Durban is at the advanced stages of climate integration and mainstreaming into municipal planning and implementation of high provides a good case and practice that can be adopted by it. This was achieved through, political commitment and oversight, and climate champions in various line departments within the city. Mather et al. [75] attribute the success in Durban to the development of high-level technical expertise within the municipality as well as advocacy by political and technical champions, and broad based and appropriate communication with stakeholders. In contrast, the city of Windhoek has an environment desk which deals with issues to do with water reclamation and recycling and use of wastewater for green spaces are imminent. While Harare Municipality has a full department in charge of waste management, the authority has no department that deals with climate related issues, which exposes the authority when it comes to environmental protection and management. In addition, although climate change adaptation strategies are being crafted at the national level in Zimbabwe through the former Ministry of Environment, Water and Climate (MoEWC), climate change adaptation has received minimum attention at the local level (Harare City Council) with no adaptation strategies in place and like most cities a department dedicated to climate and environmental issues is imminent [76].

Lessons on mainstreaming climate adaptation can be applied from the CoW that is in the process of finalizing development and approval of the Integrated City Strategy and Action Plan on climate change bearing witness of a good example of cascading the national policy on climate change to city level. Climate integration and adaptation in Durban is done in two ways: firstly, through the Durban Climate Change Strategy (DCCS), which lays out a city-wide approach to address ten interrelated climate change themes (Water, Sea level rise, Biodiversity, Food security, Health, Energy, Waste and Pollution, Transport, Economic development, and Knowledge Generation and Understanding). This mainstreaming is overseen politically by the Municipal Climate Change Committee, and administratively by the Disaster Management Advisory Forum’s Municipal Adaptation Planning Technical Task Team [77].

Secondly, the Durban Adaptation Charter (DAC) is an international agreement that commits local governments to local climate action in their jurisdiction. The DAC was launched at COP17 hosted in Durban in December 2011. In Durban, the DAC is implemented through the maintenance and advancing of the Central KwaZulu-Natal Climate Change Compact [77], which is a partnership between Durban and its surrounding local and district municipalities to collaboratively address climate change at a regional scale in an integrated fashion [77]. The DAC has coined this method as the Hub and Compact approach. The climate adaptation work of eThekwini Municipality is coordinated under the Municipal Climate Protection Programme, by the Climate Protection Branch of the Environmental Planning and Climate Protection Department.

## Discussion

### Key issues in the urban ecosystems

Water and energy vulnerability and risk are significant issues for the cities except for Durban. For instance, the city of Harare particularly is dependent on a polluted source of water (Lake Chivero) and the Kariba Dam along the Zambezi River for its electricity. In addition, the city has limited capacity to deal with increased wastewater as the infrastructure has not been adequately expanded to accommodate the population expansion [66]; [78]; [79]. This resonates with the fact that, water stress is likely to adversely impact public health, water availability, energy, forestry and biodiversity, rangelands, human settlements and tourism [62]; [3]; [70]. Recently (2019), long power cuts, especially in Lusaka and Harare, have been the order of the day with those who can afford to turning to alternative sources such as liquid gas, generators and solar power while the poor use cheaper sources of energy such as wood and kerosene [66]; [67]. Energy issues conversely affect potable water supply as water treatment processes and the pumping supply systems are reliant on electricity.

Although the quantity and quality of water supply are central to water security [62]; [63]; [69], water governance has a central role in the issue of water security. While urban agriculture supplements the city’s food demand, it has implications on water demand given that agriculture production and horticulture draws significant amounts of water for irrigation [63]; [62]. Increased populations have found homes in informal settlements, which tend to have poor quality infrastructure and amenities such as roads, electricity, water, sanitation and good transport [63].

For all the cities, there is tacit recognition that complexity of issues threatening water security in the region cannot be addressed by any one actor or one sector alone and therefore, collective leadership is required. An innovative, multi-stakeholder approach to collaboration at all levels, now enshrined in Sustainable Development Goal (SDG) 17, is emerging whereby partners increased their understanding of water risks, raise awareness of these risks and advocate for change, and harmonize resources and capacities to engage in effective, on-the-ground projects. For the city of Lusaka, the water security initiative is a case in point and in Windhoek the efficacious water reclamation systems that are governed by a PPP resonate the importance of collaboration.

### City learning points

#### Water and waste issues

The lessons and best practices of water conservation from the CoW are relevant to other FRACTAL cities such as Cape Town, Harare, Johannesburg and Lusaka, which are in the arid belt of the region and therefore susceptible to drought episodes as well as other southern African cities in the same zone [62]. This is not to say that the other cities are not acting towards water conservation, but CoW provides an excellent example of a city that is managing water scarcity well [71]. In this case cities like Harare that have a perpetual water crisis could draw lessons from CoW given the significantly higher amount of rainfall that the former receives per annum. Considering that the region has since 1960 been experiencing a decrease in annual rainfall [80] the *status quo* should not be maintained, and Lusaka and other cities in the region can learn from CoW where water conservation and wastewater reclamation is being done.

Green business opportunities at the community level can help create an enabling environment for active participation in climate change mitigation and adaptation projects [81; 82]. A key learning point from the cities of Harare, Lusaka and Durban is the concept of including the community in the cleaning which contributes towards better solid waste management. This appears to be a feasible option for Lusaka, where many actions have already formally involved communities; as well as more recently in Harare (and across Zimbabwe), where community cleaning efforts scheduled for the first Friday of every month (since November 2018) have begun to bear fruit.

#### Informal settlements

The multi-faceted and interconnected nature of several issues that cross the ecological-socio-economic divide resonate with situations across most cities in southern Africa. One lesson learnt through the exchange visits was how to address the challenge of informal settlements *vis-à-vis* building resilience in the cities as low-income households and informal households often located in unsuitable areas are most vulnerable to the compounding effects of both non-climatic and climate risks such as floods in Lusaka. The exchange visit brought out the advantages existing in CoW where temporary unplanned and illegal structures are constructed using corrugated iron sheets. These can easily be removed or moved for upgrading and planning given that most of these settlements are situated in unsuitable land.

A lesson for other southern African and African cities on dealing with informal settlements that mushroom as a result of population expansion and urbanization and the subsequent failure of service delivery can be drawn from Harare and Zimbabwe. Formalisation of informal settlements and building initiatives in Harare may provide lessons to other African cities that have problems with informal settlements and associated risks such as the famous Kibera in Nairobi, partially informal Khayelitsha township in Cape Town, and in Dar es Salaam that has an informal settlement areal coverage of 70% [83] comparable to Lusaka and to a lesser extent, Windhoek and Durban.

#### Energy issues

The City of Windhoek provides an exceptional case as the function of energy provision is designated at city level, whereas in most African countries the energy provider is often a public entity at national level. However, most southern African countries and indeed cities, which are large consumers of energy, rely on hydropower energy except for South Africa, and Botswana which mostly rely on fossil fuels, and coal in particular [84]. There is controversy over the continued use of coal in the region (with 90% of South Africa’s energy source being coal-powered), given its contribution to CO_2_ and greenhouse gases and ultimately global warming. We found comparable efforts towards use of renewable energy sources for Harare and Windhoek with projects such as the Southern African Solar Training and Demonstration Initiative (SOLTRAIN) and the developments at the SADC Centre for Renewable Energy and Efficiency (SACREE) that both Namibia and Zimbabwe are party to.

#### Water governance

Each city has taken up a water governance system which appears to be feasible and workable. There are several multiple roles of city authorities in governance which influence resilience. These include the role cities play as regulators, as taxation and licensing authorities; as strategic land-use planners and developers; consumers and providers of goods and services. This makes local authorities exceptionally well positioned to lead and influence climate adaptation interventions and stimulate behaviour change among citizens and businesses. Adaptation measures are needed in this and many cities in the region given projections of population exponential population growth.

The differing contexts of water governance demonstrated for the four cities of Harare, Windhoek, Lusaka and Durban are an example of the use of diverse solutions within African cities. Often, one size fits all solutions are advocated for and sometimes even funded for Africa, forgetting the different contextual realities. However, we acknowledge that there is still room for cities to learn from each other on implementation in order to improve on water service delivery and water resource management. Multiple actors, often through partnership, tend to intervene in urban climate change governance [81]; [82]. This suggests the use of new frameworks such as the Resilience City Planning Framework (RCPF), which is a network of interlinked multidisciplinary concepts. At the same time, governance systems in the cities need to be transparent, flexible, have integration and monitoring systems, and embrace continual learning and knowledge sharing to increase the likelihood of transformational adaptation.

#### City climate integration and planning

By engaging in compact partnerships for Durban with neighbouring municipalities, learning, resources and skills are shared and an integrated response to climate change has been developed in this city-region. This increases the scale at which work is done, often encapsulating whole catchments and substantially increasing the number of benefiting city-region residents. Lessons and good practice therefore emanated from Durban in the way in which climate change is integrated in planning processes and climate adaptation is facilitated. The structures and approaches in Durban present learning point as such efforts of integrating climate action into policy can be replicated in other southern African cities. It is worth noting though that similar efforts such as the development of Windhoek’s climate change response strategy; Lusaka’s water security initiative and some engagements on improving planning and mainstreaming national and transformative adaptation plans at the city level in Harare are all initiatives that have been borne partly from the FRACTAL engagements and from the learning experiences of the city exchanges.

## Conclusion

Cities can be regarded as complex urban ecosystems, which, like other types of ecosystems, are threatened by climate change. Additionally, southern African cities face multiple stressors and complex challenges. As with natural ecosystems, resilience against climate change lies firstly in the healthy functioning of the city and secondly in the ability of the city to adapt to changes. A crucial element in adapting is having the ability to learn and implement best practices, often from other cities facing similar challenges. As indicated in this paper, some cities have come up with innovative ways of tackling challenges such as water insecurity, informal settlements and climate change. These provide learning points for other cities that are grappling with climate change and other, similar developmental issues, also bearing in mind that context matters. In this paper we identified five elements (called ‘city learning points’) of a city that need to be considered when grappling with and building resilience to climate change impacts in southern African cities. Among these learning points are the issue of public-private partnerships, which southern African cities can embrace in dealing with issues such as water scarcity and management issues and where great strides in improving water service delivery to the city residents through water reclamation, have been made in the case of the City of Windhoek. This has placed the city on a success trajectory, which promises to lessen water availability issues that are compounded by climatic risks.

Another learning point was that community engagement and inclusion to deal more successfully with solid waste allows for local participation and green job creation in cities as evidenced by Durban and Lusaka. Community engagement ensures better understanding of the context as the solutions are made together ‘with’ rather than ‘for’ the people on the ground and who are affected by the day to day challenges. There is, however, evidence of the need for continued capacity building and improvement of the model used to implement such approaches such as in Harare’s clean up campaigns. One other critical learning point is on decisive leadership in dealing with informal settlements as exemplified by Harare. Intolerance of informal settlements and upgrading of these settlements, are strategies that promise to address criminality and disease in the low-income areas of the cities, which other cities can emulate and implement to the extent possible. Lastly, as highlighted in Durban, development and approval of city strategies and action plans on climate change adaptation provide an opportunity for cities to integrate climate change in planning processes and cascade national policies to the city level for the benefit of city residents. Mainstreaming of climate change also directly or indirectly addresses other developmental challenges such as biodiversity loss and water security.

We conclude that the evidence of efforts towards environmental protection, improved urban amenities and infrastructure and ultimately service delivery in the southern African cities studied gives an indication that to a large extent, there is scope for these cities to learn from each other to build and develop resilience. Therefore, city to city learning provides a useful platform for the exchange and uptake of ideas, practices and strategies to enhance the resilience of urban ecosystems particularly across regions such as southern Africa where similar socio-environmental issues are encountered. These lessons can certainly be useful for similar cities and regions.

## Supporting information

S1 File Windhoek-Lusaka city learning exchange questionnaire. (DOC)

S2 File Household questionnaire: Lusaka START GEC; flood occurrence, impacts and response at household level. (DOC)

S3 File Lusaka START GEC household questionnaire. (DOC)

S4 File Lusaka GEC key informants interview guide. (DOC)

S5 File Lusaka GEC focus group discussion guide. (DOC)

S6 File Harare GEC household questionnaire. (DOC)

S7 File Harare START GEC interview guide for the stakeholders. (DOC)

S8 File Agenda for FRACTAL Harare Team exchange visit, Windhoek, Namibia. (DOC)

S9 File Agenda for Harare FRACTAL Team exchange visit, Lusaka, Zambia. (DOC)

S10 File Lusaka-Windhoek visiting agenda. (DOC)
