# Supplementary material for: City to city learning and knowledge exchange for climate resilience in southern Africa
Source: PLoS One. 2020 Jan 24;15(1):e0227915. doi: 10.1371/journal.pone.0227915 (PMC6980534; doi:10.1371/journal.pone.0227915)
Supplement: S1 File — (DOC) [file pone.0227915.s001.doc]

S1 Table.

**
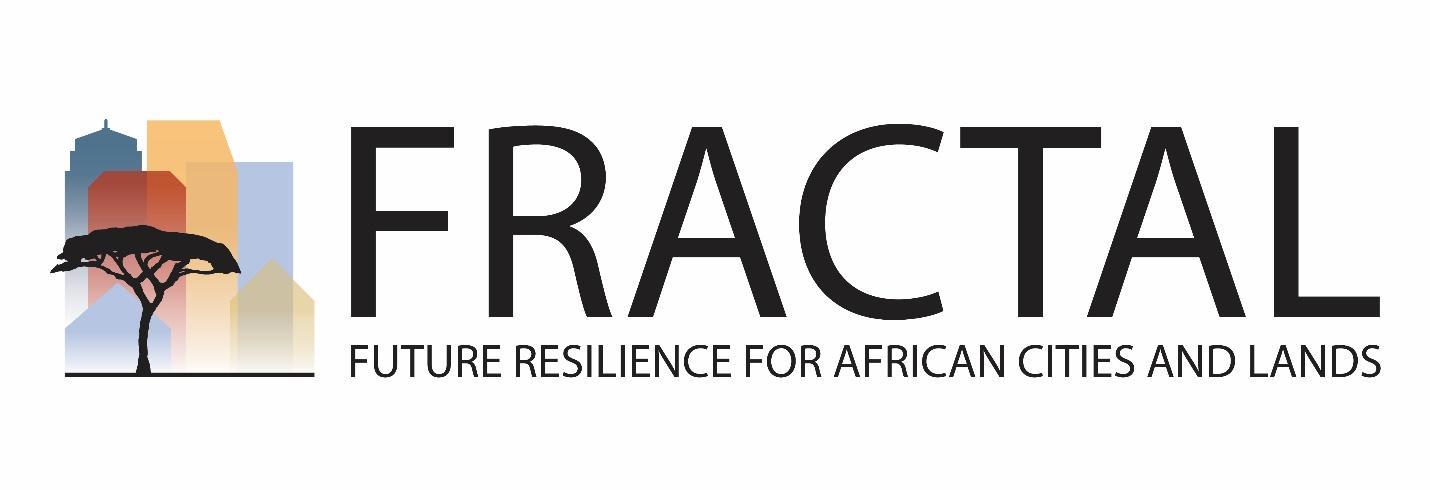
**

**Windhoek-Lusaka City Learning Exchanges Feedback Guide**

**Lusaka: 16-17 October 2017**

**Windhoek: 02-03 November 2017**

**Questionnaire**

| **DATE** |
| --- |
|  |

| **NAME** |
| --- |
|  |
| **Institution and Position** |
|  |

| **Main key lessons learnt** |
| --- |
|  |

| **Key challenges identified** |
| --- |
|  |

| **OPPORTUNITIES IDENTIFIED** |
| --- |
|  |

| **QUESTIONS I STILL HAVE (MAY HAVE ARISEN FROM DAY)** |
| --- |
|  |
