# Supplementary material for: City to city learning and knowledge exchange for climate resilience in southern Africa
Source: PLoS One. 2020 Jan 24;15(1):e0227915. doi: 10.1371/journal.pone.0227915 (PMC6980534; doi:10.1371/journal.pone.0227915)
Supplement: S2 File — (DOC) [file pone.0227915.s002.doc]

Questionnaire code___________ Interviewer name_______ Residential area__________ Date__________ GPS COORDINATES: X______________Y___________________

A. Basic household information

1. What is your gender? A. Female B. Male.
2. Household size? Below 15 years_______ Above 15________Total_______
3. How old are you? ______
4. What is your marital status?

A. single B. Married C. Separated D. Divorced E. widowed

1. What is your highest level of education?

A. None B. Primary C. Secondary D. College E. University

1. How long have you lived in this area?__________ (specify period unit)
2. Kindly estimate your current monthly household income______________
3. State the sources of you household income?
4. Ownership of house property A. Tenant B. Landlord C. Others specify ___________
5. State of house you live in. (Tick where appropriate)
6. How often do you experience floods? (Tick where appropriate)

| Frequency | Often | Rarely | Never |
| --- | --- | --- | --- |
| 2016/17 season |  |  |  |
| Frequency | Often | Rarely | Never |
| Past years |  |  |  |
|  |  |  |  |

1. Kindly tick which month the floods started and ended; also, when you experienced the worst flood incidence this year.

|  | Start | End | Worst incidence |
| --- | --- | --- | --- |
| December |  |  |  |
| January |  |  |  |
| February |  |  |  |
| March |  |  |  |
| April |  |  |  |

1. How did the floods affect you?
2. House__________
3. Roads__________
4. Waste__________
5. Water__________
6. Sanitation(toilet) __________
7. Others (specify) __________
8. How did you manage to survive through the flood effects?

|  | What did you do as a result of the flood effects? |
| --- | --- |
| House |  |
| Roads |  |
| Waste |  |
| Water |  |
| Sanitation (toilet) |  |
| Health |  |
| Income |  |
| Others (specify |  |

15. What did you do to mitigate/prevent (*ukuchingilila, kukwabililia, kuchingiliza*), against floods and their impacts?

a. As an individual____________________________________________________

b. As a community______________________________________________________

16. What challenges did you face in your efforts to mitigate against the harmful impacts of floods?

a. As an individual_________________________________________________________

b. As a community_________________________________________________________

17. What benefits were realised from floods?

A. as an individual___________________________________

b. As a community______________________________________

18. Did you receive any early warning about the potential for floods this year?

| Source of information | Channel of communication | Content | Was it helpful (how? Why?) |
| --- | --- | --- | --- |
|  |  |  |  |
|  |  |  |  |
|  |  |  |  |
|  |  |  |  |
|  |  |  |  |

19. Kindly rank the following causes of floods (1 as the most important and 6 as the least important)

| **Causes** | **Rank** |
| --- | --- |
| Solid waste (*Vinyalala*) |  |
| Poor urban planning (location of houses, roads, dump sites, open/green spaces) |  |
| Poor drainage infrastructure |  |
| Environmental Factors (High ground water table, vegetation cover, soil types) |  |
| Rainfall |  |
| Illegal land allocation |  |

- 1. For each of the following causes of floods, what do you think should be done to improve the situation by?

a. Individual households

| **Causes** | **Suggested solutions** |
| --- | --- |
| Solid waste |  |
| Poor urban planning |  |
| Poor drainage |  |
| Environmental Factors |  |
| Illegal land allocation |  |

b. Community

| **Causes** | **Suggested solutions** |
| --- | --- |
| Solid waste |  |
| Poor urban planning |  |
| Poor drainage |  |
| Environmental Factors |  |
| Illegal land allocation |  |

c. Government

| **Causes** | **Suggested solutions** |
| --- | --- |
| Solid waste |  |
| Poor urban planning |  |
| Poor drainage |  |
| Environmental Factors |  |
| Illegal land allocation |  |

d. Private sector (business)

| **Causes** | **Suggested solutions** |
| --- | --- |
| Solid waste |  |
| Poor urban planning |  |
| Poor drainage |  |
| Environmental Factors |  |
| Illegal land allocation |  |

e. Civil society

| **Causes** | **Suggested solutions** |
| --- | --- |
| Solid waste |  |
| Poor urban planning |  |
| Poor drainage |  |
| Environmental Factors |  |
| Illegal land allocation |  |
