# Supplementary material for: City to city learning and knowledge exchange for climate resilience in southern Africa
Source: PLoS One. 2020 Jan 24;15(1):e0227915. doi: 10.1371/journal.pone.0227915 (PMC6980534; doi:10.1371/journal.pone.0227915)
Supplement: S3 File — (DOC) [file pone.0227915.s003.doc]

Questionnaire code___________ Interviewer ID_______ Residential area__________ Date__________ GPS COORDINATES___________

**SECTION A**

**A. Basic Household Information**

1. What is your gender? A. Female B. Male.
2. Household size? Below 15 years_______ Above 15________Total_______
3. How old are you? ______
4. What is your marital status?

A. Single B. Married C. Separated D. Divorced E. Widowed

1. What is your highest level of education?

A. None B. Primary C. Secondary D. College E. University

1. How long have you lived in this area?__________ (specify period unit)
2. Kindly estimate your current monthly household income______________
3. State the sources of you household income? ____________________________________
4. Ownership of house property A. Tenant B. Landlord C. Others specify ___________
5. Describe of house you live in

**SECTION B**

**A. Type and Quantification of Waste**

1. Which of the following types of waste do you generate in your household?

| **Type of waste generated** | **Tick**  √ | **Estimated Quantity** | | | | |
| --- | --- | --- | --- | --- | --- | --- |
| **Paper** |  |  |  |  |  |  |
| **Plastic** |  |  |  |  |  |  |
| **Food** |  |  |  |  |  |  |
| **Sanitary waste** |  |  |  |  |  |  |
| **Bottles and cans** |  |  |  |  |  |  |
| **Debris** |  |  |  |  |  |  |
| **Others (specify)** |  |  |  |  |  |  |

2. Do you separate your waste? Yes No Reason_____________________________________________________________________

3. If the answer is yes, where do you take the separated waste? ________________________

4. How much do you pay for waste collection per month? ____________________________

If no payment, explain why? ___________________________________________________

___________________________________________________________________________

5. To whom do you pay?

- Council [ ]
- Private companies [ ]
- Private individual [ ]
- Others (specify) [ ]

6. How often do you pay?

- Daily [ ]
- Weekly [ ]
- Fortnightly [ ]
- Monthly [ ]
- Upon Collection [ ]

7. How often is waste collected from your home by the contracted party?

- Daily [ ]
- Weekly [ ]
- Fortnightly [ ]
- Monthly [ ]
- More than a month [ ]
- Never [ ]

8. If waste is never collected, how do you dispose of your waste? (Tick what is applicable)

- Pit [ ]
- Burn [ ]
- Toilet [ ]
- Council dumpsite [ ]
- Others (specify) [ ]

9. Kindly state your views on the formal dumping sites in terms of the following aspects

1. Distance…………………………………………………………………………………………………………………………………………………………………………….
2. Waste Collection……………………………………………………………….............

………………………………………………………………………………………….

1. Waste Collection Fees………………………………………………………….............

………………………………………………………………………………………….

10. What action should be taken to improve on waste collection by:

A. Government/ Council ……………………………………………………………………

……………………………………………………………………………………………………………………………………………………………………………………………………

B. Community…………………………………………………………………………………..

……………………………………………………………………………………………………………………………………………………………………………………………………

C. Individuals…………………………………………………………………………………...

………………………………………………………………………………………………….
