# Supplementary material for: City to city learning and knowledge exchange for climate resilience in southern Africa
Source: PLoS One. 2020 Jan 24;15(1):e0227915. doi: 10.1371/journal.pone.0227915 (PMC6980534; doi:10.1371/journal.pone.0227915)
Supplement: S4 File — (DOC) [file pone.0227915.s004.doc]

**Section A: Solid waste management system**

1. What are your institutional day to day roles/profile of activities and who are your partners?
2. How is your institution involved in flood risk reduction and preparedness in Lusaka?
3. Does your institution focus on a particular role or many in (a). mitigating against floods? (b). Solid waste management
4. What are your views / experiences on the performance solid waste management through public private partnerships?
5. How to you assess the informal players (‘scavengers’ and individual solid waste collectors from homes’).
6. How would you describe the use of climate related information in your institution’s daily activities?
7. Based on your role in disaster preparedness and response, how does solid waste contribute to urban flooding in Lusaka? (also perceived linkages between solid waste management and flood)
8. How would you describe the current and passed solid waste management systems in relation to urban flooding in Lusaka?
9. Based on your understanding of the entire solid waste management chain, where in this chain do you think there exists major flaws? Use examples.
10. Out of a ten point scale, how would you rate or describe the existing solid waste management systems in Lusaka?
11. What are the decision-making systems and platforms for solid waste management?
12. How do you think the solid waste management in Lusaka can be improved?
13. What are the necessary factors or conditions for success in solid waste management in Lusaka?

**Section B. Flood Risk Reduction and Management**

1. Based on your institutional roles in mitigating against floods and flood effects in Lusaka, how does the nature of urban planning and land use contribute to urban flooding in informal settlements?
2. How does infrastructure planning, development and management (roads, drainage, and solid waste management) imped/enhance your efforts to mitigate against flood occurrence and flood risk?
3. How does your institution integrate solid waste management and infrastructure planning in mitigating against floods and flood risk in informal settlements in Lusaka?
4. How does your role get integrated in the Lusaka district disaster response framework?
5. What are the decision-making systems and platforms for flood risk reduction and response?
6. What are the key considerations in decision-making to mitigate against floods and flood risk?
7. How have these platforms shaped or influenced flood disaster preparedness and response over since 2000? Use specific examples to explain your answer.
8. What mechanisms are used to disseminate information and decisions about flood disaster preparedness and response?
9. Is there any discrepancy between the decisions made and the actions taken to mitigate against floods?
10. Why does this discrepancy exist?
11. How does the current and passed institutional arrangements affect flood mitigation and flood risk reduction?
12. What improvements would propose to improve institutional performance to mitigate against floods and flood risk?
13. Given your experiences, which actor would you recommend to assume the coordination role in mitigating against flood occurrence and flood risk reduction?
14. What are your final reflections on the subject? (way forward?)

Thank you!
