# Supplementary material for: City to city learning and knowledge exchange for climate resilience in southern Africa
Source: PLoS One. 2020 Jan 24;15(1):e0227915. doi: 10.1371/journal.pone.0227915 (PMC6980534; doi:10.1371/journal.pone.0227915)
Supplement: S5 File — (DOC) [file pone.0227915.s005.doc]

**PART 1-ASSESMENT OF THE CURRENT STATE**

**A. FLOOD MANAGEMENT SYSTEMS**

|  | **Problem context** | | **Flood management systems** | | | | | | **Action points** |
| --- | --- | --- | --- | --- | --- | --- | --- | --- | --- |
| Period-2016-17 | Prone area | Causes of floods | Response system | Actors/ structures | Roles | Coordination at various scales | Success | Failures | Way forward |
|  |  |  |  |  |  |  |  |  |  |
|  |  |  |  |  |  |  |  |  |  |
|  |  |  |  |  |  |  |  |  |  |
|  |  |  |  |  |  |  |  |  |  |
|  |  |  |  |  |  |  |  |  |  |
|  |  |  |  |  |  |  |  |  |  |

**B. SOLID WASTE**

|  | **Problem context** | | **Solid waste management systems** | | | | | | **Action Points** |
| --- | --- | --- | --- | --- | --- | --- | --- | --- | --- |
| Period-2016-  17 | Areas with volumes of solid waste | Causes of waste accumulation | Waste disposal system | Actors/  structures | Roles | Coordination at various scales | Success | Failures | Way forward |
|  |  |  |  |  |  |  |  |  |  |
|  |  |  |  |  |  |  |  |  |  |

**C. Necessary factors /conditions for change for the better-Perquisites for change for the better (reflections on the roles/practices by various actors)**

**PART 2: ASSESEMENT OF CHANGES**

**D. FLOOD MANAGEMENT SYSTEMS**

| **Problem context** | | **Flood management systems** | | | | | | **Action points** |
| --- | --- | --- | --- | --- | --- | --- | --- | --- |
| Prone area | Causes of floods | Response system | Actors/  structures | Roles | Coordination at various scales | Success | Failures | Way forward |
|  |  |  |  |  |  |  |  |  |
|  |  |  |  |  |  |  |  |  |
|  |  |  |  |  |  |  |  |  |
|  |  |  |  |  |  |  |  |  |
|  |  |  |  |  |  |  |  |  |
|  |  |  |  |  |  |  |  |  |
|  |  |  |  |  |  |  |  |  |

**E. SOLID WASTE IN HISTORY**

| **Problem context** | | **Solid waste management systems** | | | | | | **Action Points** |
| --- | --- | --- | --- | --- | --- | --- | --- | --- |
| Prone area | Causes of waste accumulation | Waste disposal system | Actors/  structures | Roles | Coordination at various scales | Success | Failures | Way forward |
|  |  |  |  |  |  |  |  |  |
|  |  |  |  |  |  |  |  |  |
|  |  |  |  |  |  |  |  |  |
|  |  |  |  |  |  |  |  |  |

**PART 3: LESSONS LEARNT (PLENARY DISCUSSIONS)**

1. Intuitional coordination
2. Policy guide
3. Practice
4. Role of the government
5. Role of the council
6. Role of Politicians
7. Role of private sector
8. Planning in the face of flood related risks and disasters
9. Suggested framework of action
