# Supplementary material for: City to city learning and knowledge exchange for climate resilience in southern Africa
Source: PLoS One. 2020 Jan 24;15(1):e0227915. doi: 10.1371/journal.pone.0227915 (PMC6980534; doi:10.1371/journal.pone.0227915)
Supplement: S6 File — (DOC) [file pone.0227915.s006.doc]

Interviewer………………………… Date…………………………………..

Suburb…………………………....... GPS points……………………………

Start time…………………………….. End time……………………………

**Section A: Demographic Data**

| 1. Location   1*. Mabvuku*  *2. Stonridge*  *3. Mainway Meadows*  *4. Malbrough*  *5. Mt Pleasant Heights*  *6.Chisipite/Vinona* | 1. Sex of household head?   *1. Male 2. Female* | 1. Name of household head 2. Name of respondent | 1. Relationship with the Household Head 2. *Self* 3. *Child* 4. *Spouse* 5. *Other*______ |
| --- | --- | --- | --- |

**Section B: Climate**

| 1. Have you ever heard of climate change?  *1.Yes*  *2. No* | 2. If so, from what source?  *1.T.V, 2. Radio, 3. Newspaper, 4. Internet*  *5. Councillor, 6. Friend/Relative, 7. Self-observation, 7. Work place*  *8. Other*  *Specify…………………………….* |
| --- | --- |
| 3. Have you ever noticed any significant changes in rainfall over the years?  *1.Yes, 2. No* | 4. If yes, what are the changes?  *1. Increase, 2. Decrease, 3. Other observations*  When did you experience the changes? |
| 5. Have you ever noticed any significant changes in temperature over the years?  *1.Yes, 2. No* | 6. If yes, what are the changes?  *1. Increase, 2. Decrease, 3. Other observations* |
| 1. In which year/years did you experience noticeable temperature changes?   *Decrease..................................................................................................................................*  *Increase…………………………………………………………………………………………………* | |
| 8. What other changes in climate have you noticed? ..............................................................................  ………………………………………………………  ………………………………………………………  …………………………………………………………………………………………………………... | 9. Have you ever experienced any loss as a result of climate related events?  *1.Yes, 2. No* |
| 10. If so what are the losses you have experienced?  *1. Damage to property*  *2. Damage of horticultural crops*  *3. Other specify…………………………………………………………………………………………………………………* | 11. Have ever experienced deaths/ illness in the family as a result of climate change related hazards/ events?  *1. Yes*  *2. No* |
| 12. If yes, describe deaths/illness  …………………………………………………………………………………………….. | 13 Are there any other challenges that you face in relation to climate change?   1. *Yes, 2. No* |
| 14 Are there any organisations, stakeholders or individuals which have come to your assistance in the event of climate change related hazards?   1. *Yes 2. No* | |
| 15.If yes, which organisations, stakeholders, individuals are these  *1. NGOS, Specify…………………………………………………………………………………………………..,*  *2. Government, specify………………………………….…………………………………………………………..*  *3. Community leaders e.g. Councillors, 4. Friends, 5. Relatives,*  *6. Other, specify……………………………………….* | |
| 16. Do you have any adaptation/coping strategies that you employ in relation to climate change challenges you have mentioned?  1. *Yes 2. No* | 17. What are the adaptation/coping strategies?  .....................................................................  ……………………………………………  ……………………………………………  ………………………………………………………………………………………… |

**Section C: Climate/Weather Information**

| 1**.** What type of weather information do you receive?  1*. Temperature, 2. Rainfall, 3. Wind, 4. Humidity 5. Sunshine, 6. Pressure* | | 2.Through what channel do you receive the of information?  Channel Source  *1. SMS*  *2. Internet*  *3. Schools,*  *4. Neighbour,*  *5. Radio,*  *6. Television,*  *7. Newspapers*  *8. Don’t receive*  *9. Other (specify)* |
| --- | --- | --- |
| 3. When do you receive it?    *1 Daily, 2 Weekly, 3 Monthly, 4 Quarterly (state months), Other****__*** | | 4. Upon receiving this information, what action do you take?  ---------------------------------------------------------- |
| 5. How would you rate the information you receive based on the following?  Rating  *Timeliness - 1 Poor, 2 Average, 3 Good*  *Frequency of dissemination - 1 Poor, 2 Average, 3 Good*  *Usefulness - 1 Poor, 2 Average, 3 Good*  *General content - 1 Poor, 2 Average, 3 Good*  *Language of presentation - 1 Poor, 2 Average, 3 Good* | | |
| 6. Are there any organisations, stakeholders or individuals which assist you with information on climate change risks and adaptation strategies?  1. *Yes*  *2. No* | 7. If yes, which organisations are these  *1. NGOS, Specify…………………………………………………………………………………………………..*  *2.Government,*  *Specify……………………….……………………………………………………………………………………..*  *3.Community leaders e.g. councillors, 4. Friends, 5. Relatives*  *6. Other,*  *specify……………………………………….* | |

**Section D: Water**

| 1. Do you have access to water resources in your suburb?  *1.Yes, 2. No*  *If answer is no, move to number 8* | | 2. If yes, which water sources do you use?  *1. Council, 2. Borehole, 3. Protected well*  *4. Unprotected well, 5. River*  *6. Buy water from external source*  *7. Other, specify………………………………………* | |  |
| --- | --- | --- | --- | --- |
| 3. If it is council, how many days do you get water supply per week?  *1. Everyday*  *2. 4 – 6 days*  *3. 2 – 4 days*  *4. Once* | | 4. If it is not tap water, what distances do you travel for your water supply?  *1. Less than 100 metres, 2. 100-500 metres*  *3. 600- 1000 metres, 4. 1 km to 1,5 km*  *5. More than 1,5 km* | |  |
| 5. How much time do you spend fetching water?  *1. Less than 30 minutes,*  *2. 31-60 minutes*  *3. 1 hour to 1 hour 30 minutes,*  *4. 1 hour 30 to 2 hours,*  *5. More than 2 hours* | | 6. Approximately how many other households get water from the same source?  *1. Less than 10, 2.11 to 20, 3. 21-30*  *4. More than 30* | |  |
| 7. Who is responsible for fetching water in this house hold?  *1. Father, 2. Mother, 3. Male child, 4. Female child, 5. Domestic worker* | | 8. Do you think that the water you are getting is safe? *1.Yes, 2.No* | |  |
| 9. Have you ever had any illness that you perceive to be as a result of the water you use/water borne diseases?  *1.Yes, 2. No* | | 10. If so, which ones  *1. Typhoid, 2. Cholera, 3. Diarrhoea, 4. Bilharzia*  *5. Others, Specify………………………..* | |  |
| 11. Do you ever treat your water you have access to before using it?  *1.Yes, 2. No* | | 12. What method do you use to treat your water?  *1. Boil, 2. Chemical treatment (Specify)*  *3. Filtration*  *4. Other…………………………….………………* | |  |
| 13. Do you have access to communal sources of water?  *1. Yes*  *2. No*  *3. Do not know* | | 14. Who installs/maintains the water source? ......................................  *1. NGOS, Specify………………………………………*  *…………………………………………………………..*  *2. Community leaders e.g. councillors*  *3. Government, specify………………………………………………*  *4.Others, specify……………………………………* | |  |
| 15. In case of water shortages, do you have any organisations or stakeholders you can report to for assistance?  *1. NGOS, Specify…………………………………….*  *…………………………………………………………..*  *2.Community leaders e.g. councillors*  *3. Government, Specify………………………………….…………………………………………………………………………..*  *4. Others,*  *Specify………………………………………* | | 16. In the case of water shortages have you ever reported to the any of those organisations?  *1. Yes*  *2. No* | |  |
| 17. Do you get assistance when you report?  *1.Yes*  *2.No* | | 18. If yes, what kind of assistance?  *………………………………………………………………………………………………………………………………………………………………………* | |  |
| 19. If not, do you get reason as to why?  …………………………………………….........  ………………………………………………….  …………………………………………………. | | 20. What other water supply related problems do you face?  ……………………………………………….  ……………………………………………….  ……………………………………………….. | |  |
| 21. What has been the trend in water supply for the last 30 years?  Answer 1, 2 or 3 = *1. Increasing, 2. Decreasing 3.No change*   | ***Years*** | ***Trend*** | | --- | --- | | 1-5 |  | | 6-10 |  | | 11-15 |  | | 16-20 |  | | 21-25 |  | | 26-30 |  | | | | |  |
| 22. What changes in water supply have you noticed in the past 30 years? In which years have you noted the change?   | **Change** | **Years** | **Cause** | | --- | --- | --- | | *Increasing* |  |  | | *Decrease* |  |  | | *No change* |  |  |   23. What do you think could have been the cause? *Answer in the table in No. 22*  1. *Climate change*  *2. Governance systems*  *3. Population increase*  *4. Natural causes*  *5. Other, specify* | | | |  |
| 24. Besides water supply, what other water related problems do you face?  ……………………………………………….  ……………………………………………….  ………………………………………………..  ………………………………………………..  ………………………………………………… | | 25. Are there any meetings that address water issues in your community which are held with any stakeholders involved with water supply in your community?  *1. Yes*  *2. No*  *3. I do not know* | |  |
| 26. If yes, do you attend those meetings  1.Yes, 2. No | | 27. If yes, how often?  *1. Once a week, 2. Once a month, 3. Twice a month, 4. Once a year, 5. Twice a year, 6. Not at all, 7. Not applicable (I do not know) 8. Other, specify…………………………………..* | |  |
| 28. Which are the organisations, stakeholders or individuals which call for those meetings?  *1. NGOS, Specify………………………………………………………………………………………………….*  *2. Government, specify…………………………………………………………………………………………….*  *3.Community leaders e.g. Councillors*  *4. Others, specify…………………………………………* | | | |  |
| 29. Which organisation do you think is the most important in the provision of water?  *1.Council*  *2. NGOS, Specify………………………………………………………………………………………………….*  *3.Community leaders e.g. councillors*  *4. Government, specify…………………………………………………………………………………………….*  *5. Others, specify…………………………………………* | | | |  |
| **Institution/organization** | **Role /services provided** | | **Frequency of training*** | |
|  |  | |  | |
|  |  | |  | |
|  |  | |  | |
|  |  | |  | |
|  |  | |  | |
|  |  | |  | |

********1= monthly, 2= quarterly, 3= twice a year, 4= annually, 5 = ad hoc*

**Section E: Energy**

| 1. List the sources of energy that you use in in order of importance,  *1. Firewood, 2. Electricity, 3. Solar, 4. Gas*  *5.Liquid fuel e.g. paraffin, 6. Biogas, 7. Other, Specify………………………………………* | 2. Do you ever experience power cuts from the national grid supply?  1*.Yes*  *2. No* |
| --- | --- |
| 3. If yes, what is your alternative source of energy?  *1. Firewood, 2. Solar, 3. Gas, 4. Fuel e.g. paraffin*  *5. Biogas, 6. Other, specify…………………………* | 4.From the national grid, what has been the trend in energy supply for the last 30 years  Change Years  1. *Poor*  *2. Good*  *3. Excellent* |
| 5. What do you think is the cause for that change  1. *Climate change*  *2. Governance systems*  *3. Population increase*  *4. Natural causes*  *5. Other, specify* ……………………………………………………………………… | 6. Are you aware of the organisations responsible for supplying you with electricity?  1. *Yes, 2. No* |
| 7. If yes, which are those?  ……………………………………………………. | 8. In case of power cuts and faults, are there any organisations you can report to for assistance?  *1. NGOS, Specify…………………………………………………………………………………………*  *2.Community leaders e.g. Councillors*  *3.Government, Specify………………………………………………………………………………………………….*  *4. Others, specify…………………………………………* |
| 9. Do you report to any of those organisations?  1. *Yes*  *2. No* | 10. Do you get assistance when you report?  1. *Yes*  *2. No* |
| 11. If yes, in what form?  …………………………………………….........………………………………………………….  ………………………………………………….………………………………………………….  ………………………………………………….…………………………………………………. | |
| 12. What other energy related problems do you face?  ……………………………………………….  ……………………………………………….  ………………………………………………..  ………………………………………………..  ………………………………………………… | 13. Are there any meetings which are held with any stakeholders involved with energy supply in your community?  *1. Yes*  *2. No*  *3. I do not know* |
| 14. If yes, how often are they held?  *1. Once a week, 2. Once a month, 3. Twice a month*  *4. Once a year, 5. Twice a year, 6. Not at all*  *7. Not applicable (I do not know)*  *8. Other, specify*…………………………………..  …………………………………………………… | 15. Which are the organisations that organise those meetings?  *1. ZESA/ZERA*  *2. NGOS, Specify…………………………………*  *…………………………………………………*  *3. Community leaders e.g. councillors*  *4. Govenment, specify……………………………*  *5. Others, specify…………………………………* |
| 17. Do you attend those meeting?  1.Yes 2. No | 19. Which organisation do you think is the most important in the provision of energy?  *1. ZESA*  *2. NGOS Specify………………………………………*  *3. Community leaders e.g. councillors*  *4. Govenment, specify……………………………*  *5. Others, specify………………………………* |
| 16. Why do you think this is the most important in the provision of energy?  *1. ZESA/ZERA*  *2. NGOS, specify………………………………………*  *…………………………………………………*  *3. Community leaders e.g. councillors*  *4. Govenment, specify…………………………………*  *………………………………………………*  *5. Others, specify………………………………* |  |

| **Institution/organization** | **Role /services provided** | **Frequency of training*** |
| --- | --- | --- |
|  |  |  |
|  |  |  |
|  |  |  |
|  |  |  |
|  |  |  |
|  |  |  |

********1=monthly, 2=quarterly, 3=twice a year, 4=annually, 5 = ad hoc*

**Section F: Household capital endowment**

| 1. Which of the following natural capital do you have access to?  *Land*  *Water for production*  *Biodiversity (forests, trees)*  ***1= Yes 2= No*** | | 2. What are the changes in quantity over time?  *Land*  *Water for production*  *Biodiversity (forests, trees)*  ***1= No Change, 2= Increased, 3= Decreased*** | |
| --- | --- | --- | --- |
| 3. Which of the following financial capital do you have access to?  *Savings schemes, Microfinance/Credit schemes*  *Remittances, Formal employment Informal employment Cash from field crops Cash from horticultural crops, Cash from livestock sales*  ***1=Yes 2=No*** | | 4. What are the changes in quantity over time?  *Savings schemes, Microfinance/Credit schemes*  *Remittances, Formal employment Informal employment Cash from field crops Cash from horticultural crops, Cash from livestock sales*  ***1= No Change, 2= Increased, 3= Decreased*** | |
| 5. Which of the following physical capital do you have access to?  *Radio, Television, DStv decoder, Refrigerator, Deep freezer*  ***1= Yes 2= No*** | | 6. What are the changes in quantity over time?  *Radio, Television, DStv decoder, Refrigerator, Deep freezer*  ***1= No Change, 2= Increased, 3= Decreased*** | |
| 7. Which of the following is true of your human capital?  *People that can read and write Years of education*  *Skills in various activities, Health – Disease prevalence in the past 3 months, Death in the past 12 months.* ***1= Yes 2= No*** | | 8. What are the changes in quantity over time?  *People that can read and write Years of education*  *Skills in various activities, Health – Disease prevalence in the past 3 months, Death in the past 12 months*  ***1= No Change, 2= Increased, 3= Decreased*** | |
| 9.Which of the following social capital do you have access to?  *Interaction with neighbors*  *Interactions with stakeholders*  *Various clubs*  *Involvement in community meetings*  *Church groupings*  *Group savings (Mukando)*  *Burial societies*  *Market networks*  *1=Yes 2=No* | 10.What is the frequency of meetings per month?  *Interaction with neighbors*  *Interactions with stakeholders*  *Various clubs*  *Involvement in community meetings*  *Church groupings*  *Group savings (Mukando)*  *Burial societies*  *Market networks*  *1=once a week, 2=once a month 3=twice a month, 4=thrice a month 5=other* | | 11.What are the changes in number of meetings per month?  *Interaction with neighbors*  *Interactions with stakeholders*  *Various clubs*  *Involvement in community meetings*  *Church groupings*  *Group savings (Mukando)*  *Burial societies*  *Market networks*  *1=No Change, 2=Increased, 3=Decreased)* |

**Section G: Household Information**

| 1. Age of the respondent  *1. Below 30, 2. 31-40, 3. 41-50, 4. 51-65 5. Above 60* |
| --- |
| 2. How long have you been staying in this area?  *1. Less than 10 years, 2.10- 20years 3. 21—30 years, 4. More than 30 years* |
| 3. What is the age of the household head? ……....... |
| 4. Marital status of household head  *1. Single, 2. Married, 3. Divorced/Separated, 4. Widowed* |
| 5. Occupation of household head  *1. Student, 2. Self employed, 3. Unemployed, 4. Pensioner, 5. Employed/Professional*  *Specify name of profession…………….* |
| 6. Size of household  *1. 1-4, 2. 5-8, 3. 9-12, 4. Above 12* |
| 7. How may dependants do you have in this household?  *1. 1-4, 2. 5-8, 3. 9-12, 4. Above 12* |
| 8. Do any of your dependants go to school?  1. *Yes, 2. No* |
| 9. Are there any organisations which assist you in paying for your dependants’ school fees?  1. *Yes, 2. No* |
| 10. Education level of the household head  *1. Primary, 2. Secondary, 3. Vocational training, 4. Tertiary level, 5. None* |
| 11.What is your main source of income?  1*. Salary, 2. Donors, 3. Pension, 4. Gardening, 5. Vending, 6. Small business*  *7. Other, specify…………………………………..* |
| 12. What is your total income  1. *Less $100, 2. $200 - $300, 3. $301- $400, 4. $401 - $500, 5. More than $500* |
| 13.Wealth category  1. *Rich, 2. Average, 3. Poor* |
| 14. Do you sometimes get financial assistance from your children or relatives?  1. *Yes, 2. No* |
