# Supplementary material for: City to city learning and knowledge exchange for climate resilience in southern Africa
Source: PLoS One. 2020 Jan 24;15(1):e0227915. doi: 10.1371/journal.pone.0227915 (PMC6980534; doi:10.1371/journal.pone.0227915)
Supplement: S7 File — (DOC) [file pone.0227915.s007.doc]

**General**

1. Organisation
2. Your role within your organization

**Water/energy sector (depending on relevance of the informant)**

1. Access to resource in the city
2. Trends in access/availability in the city
3. Constraints to access (does climate come in)
4. Constraints/opportunities for access
5. Risk and vulnerabilities for the sector
6. Impacts on sector for households and communities
7. Strategies to deal with the risks and vulnerabilities

**Climate change**

1. Awareness of climate change
2. Climate change risks in Harare
3. Trends in climate in the city
4. Impacts in the city
5. Adaptation at organizational level
6. Adaptation at household level
7. Constraints to adaptation at various levels
8. Opportunities to adaptation at various levels
9. Impacts on operations
10. Climate change awareness and effects within the organisation

**Climate change/weather information**

1. Access to climate change information
2. Type of information and channels used
3. Climate change information organizational planning
4. Comment on information received

**City stakeholders**

1. City governance landscape (who are the stakeholders and role they play)
2. Partner organizations climate change information and adaptation
3. Collaboration and adaptation
4. Climate change as an issue among sectors
5. Climate change and policies and constraints/opportunities
6. Community outreach programmes (general and climate oriented)
