# Supplementary material for: City to city learning and knowledge exchange for climate resilience in southern Africa
Source: PLoS One. 2020 Jan 24;15(1):e0227915. doi: 10.1371/journal.pone.0227915 (PMC6980534; doi:10.1371/journal.pone.0227915)
Supplement: S8 File — (DOC) [file pone.0227915.s008.doc]

**
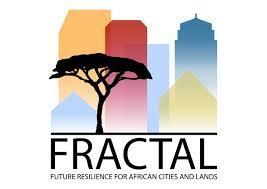
**

**Future Resilience for African CiTies and Lands Project**

**Harare Team hosted by City of Windhoek and University of Namibia**

**21-23 August 2017, Windhoek, Namibia**

**Agenda**

| Time | Activity | Facilitator | Venue |
| --- | --- | --- | --- |
| **DAY 1: 21 August 2017** | | | |
| 09:00-14:00 | Visit to Namibia Energy Institute | Kornelia, David Muchadenyika, Julia Pelaez Avila, Fenni Shidhika and Mr. Shihepo |  |
| 12:00-12:30 | GEC CUT overview: Harare | Dr. Mzime | W100, Science Block, UNAM |
| 12:30-13:00 | GEC UNAM overview: Windhoek  Components:   - Governance research - Drought research - Water and livelihoods research | Prof. Mfune | W100, Science Block, UNAM |
| **DAY 2: 22 August 2017** | | | |
| 09:00-14:00 | Site visit to Goreangab reclamation plant | Mr. James | Gorenagab, Katutura |
| **DAY 3: 23 August 2017** | | | |
| 10:30-11:00 | City of Windhoek overview on water-energy management strategies | Mr. Makuti from Environmental Management division, City of Windhoek | W100, Science Block, UNAM |
