# Supplementary material for: City to city learning and knowledge exchange for climate resilience in southern Africa
Source: PLoS One. 2020 Jan 24;15(1):e0227915. doi: 10.1371/journal.pone.0227915 (PMC6980534; doi:10.1371/journal.pone.0227915)
Supplement: S9 File — (DOC) [file pone.0227915.s009.doc]

**
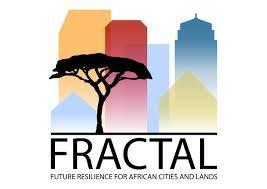
**

**Future Resilience for African CiTies and Lands Project**

**Harare Team hosted by City of Lusaka and University of Zambia**

**25-26 September 2017, Lusaka, Zambia**

**Agenda**

| Time | Activity | Facilitator | Venue |
| --- | --- | --- | --- |
| **DAY 1: 25 September 2017** | | | |
| 09:00-9:30 | GEC CUT overview: Harare | Dr. Mzime | Geography departmental library |
| 9:30-10:30 | GEC UNZA overview: Lusaka  Components:   - Interaction of planning, flooding and municipal solid waste in the city of Lusaka - Governance research in water and energy sectors in the city of Lusaka - Lessons learned | Dr. Siame, Dr. Nchito  Ms. Mwalukanga  Mr. Namutoka | Geography departmental library |
| 10:30-11:00 | Water and informality in the city of Lusaka | Dr. Siame/ Ms. Mwalukanga | Geography departmental library |
| 11:30-12:30 | Overview of Harare and GEC study: risks and vulnerabilities in water and energy sector and decision-making | Dr. Mzime and Dr. Chipo | Geography departmental library |
| 12:30- 14:00 | LUNCH | | |
| 14:00-14:30 | Curtesy visit to the Lusaka Mayor | Ms. Mwalukanga | Lusaka City Council |
| 15:00- 16:00 | Reflections of the day | Dr. Siame | Geography departmental library |
| **DAY 2: 26 September 2017** | | | |
| 08:30 | Departure for Kanyama Water Trust | All | UNZA Car Park |
| 09:30 | Arrival at the Kanyama Water Trust | Councillor Kanyama ward/Water Trust Manager | Kanyama |
| 10:00 | Tour of the settlement | All | Kanyama |
| 13:00 | LUNCH | | |
| 14:30- 15:30 | Team reflections | All | Geography departmental library |
