# Supplementary material for: City to city learning and knowledge exchange for climate resilience in southern Africa
Source: PLoS One. 2020 Jan 24;15(1):e0227915. doi: 10.1371/journal.pone.0227915 (PMC6980534; doi:10.1371/journal.pone.0227915)
Supplement: S10 File — (DOC) [file pone.0227915.s010.doc]

**
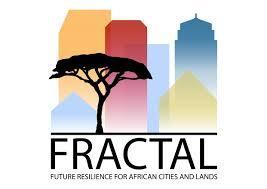
**

**Future Resilience for African CiTies and Lands Project**

**Lusaka Team hosted by City of Windhoek and University of Namibia**

**01-03 November 2017, Windhoek, Namibia**

**Agenda**

| **Time** | **Activity** | **Facilitator** | **Venue** |
| --- | --- | --- | --- |
| **Day 1: 01 November 2017** | | | |
| Arrival of Lusaka team at Hosea Kutako International Airport at 20:40  Shuttle takes team to Arebbusch Travel Lodge (Windhoek) | | | |
| **DAY 2: 02 November 2017** | | | |
| 08.00-08.15 | Introductions and welcome remarks | Prof. J Mfune, UNAM | Arebbusch Travel Lodge |
| 09:00-11:00 | Trip to Goreangab Wastewater Reclamation Plant | Dr. T Honer, WINGOC | Goreangab, Katutura |
| 11:30-13:00 | Site visit to Artificial aquifer recharge borehole | Ms. Z Scheepers, City of Windhoek | Intersection of the B2 and Frankie Fredericks drive, Olympia |
| 13:00-14:00 | Lunch | All | Arebbusch Travel Lodge |
| 14:00-15:00 | Water security in Windhoek START GEC Project | UNAM & NamWater | University of Namibia  Science Building W100 |
| 15:30-17:00 | Trip to Havana Big Bend | Ms. C Mwilima, City of Windhoek | Havana, Katutura |
| **Day 3: 03 November 2017** | | | |
| 08.30:09.30 | Discussions by City of Windhoek on water and climate change related issues | Mr. O Makuti, City of Windhoek | Head Office, City of Windhoek |
| 09.30-09.45 | Closing remarks | Mr. F Hambuda, Strategic Executive, City of Windhoek | Head Office, City of Windhoek |
| 10:00-11.00 | Lunch | All |  |
| 11:30 Departure of Lusaka team at Hosea Kutako International Airport | | | |
